# Impacts of resident physician unionization on house staff compensation

**DOI:** 10.1371/journal.pone.0308100

**Published:** 2024-10-03

**Authors:** Sidharth Tyagi, Rema J. Shah, Joshua Huttler, Jehanzeb Kayani, Mohammad-Reza Ghovanloo, Philip R. Effraim

**Affiliations:** 1 Yale School of Medicine, New Haven, CT, United States of America; 2 Department of Medicine, Icahn School of Medicine at Mount Sinai, New York, NY, United States of America; 3 Department of Neurology, Yale School of Medicine, New Haven, CT, United States of America; 4 Department of Anesthesiology, Yale School of Medicine, New Haven, CT, United States of America; Beaumont Hospitals: Beaumont Health, UNITED STATES OF AMERICA

## Abstract

**Background:**

Physicians-in-training in the United States work long hours for relatively low wages. In response to increased economic burden, the popularity of unionization in residency training programs has increased dramatically. In this study, we conducted a cross-sectional investigation of the association between unionization status and Internal Medicine PGY-1 compensation and benefits.

**Methods and findings:**

We compiled residency salary and benefits data from all Internal Medicine residency training programs in the United States. Using a mixed effects modeling approach, we evaluated the differences in salary and total compensation while adjusting for regional factors and cost-of-living differences. In aggregate, PGY-1 salary was higher for unionized vs. non-unionized programs ($69648 vs. $62214; [95% CI 670.7–3563.7]). However, there was no difference after adjusting for cost-of-living ($62515 vs $62475; [95% CI. -1317.5, 1299.7]). Unionized programs do however offer greater monetary benefits in the form of stipend disbursements, and total compensation is higher in unionized vs. non-unionized residency programs ($65887 vs $63515; [95% CI 607.6, 3551.5]).

**Conclusions:**

Unionized residency programs offer higher total compensation packages than their non-unionized counterparts. This increase in compensation is driven in large part by an increased variety and amount of stipend disbursement.

## Introduction

Physicians-in-training in the United States work long hours for relatively low wages. High rates of inflation, increases in housing costs, healthcare staffing limitations and challenges associated with the COVID-19 pandemic have intensified both economic and non-economic challenges facing resident physicians and led to discussions regarding unionization [1, 2]. Through collective bargaining, unions can advocate for increased salary and improved benefits. Across the United States workforce, those who are members of labor unions make 18% more than their non-union counterparts [3]. However, the association between unionization and internal medicine resident compensation has not been recently examined. To evaluate the influence of unionization on residency compensation, we conducted an investigation of the association between unionization status and Internal Medicine PGY-1 compensation and benefits. We chose to analyze PGY-1 compensation because residency programs scale pay based on the post-graduate year (PGY) of trainees, with the PGY-1 year representing the minimum compensation that house staff will earn during residency.

## Methods

### Study design

To compare compensation patterns in unionized vs. non-unionized residency programs, we conducted a cross-sectional analysis of United States Internal Medicine residency program compensation.

### Data sourcing

To get a comprehensive list of all US Internal Medicine Residency programs, we downloaded all programs indexed on the American Medical Association’s FREIDA database. Importantly, FRIEDA contains salary and benefit information as well, but this information was frequently inaccurate. We systematically searched program websites and residency handbooks to generate a database with accurate salary and benefit information. We determined Union Status by keyword search and manual review and cross-referenced our manually generated list with the list provided by CIR/SEIU, the largest resident union. Union dues were determined directly from union contracts. We gathered salary and benefits information from program websites and residency handbooks by manual review. If discrepancies existed between two sources of information from the same program, we took values from the more up-to-date document. We determined unionization status by keyword search and manual review of union contracts.

### Eligibility criteria

We identified 625 total Internal Medicine residency programs. We excluded all military programs (as these would not be affected by Unionization) and any programs in Puerto Rico because these programs offer significantly lower compensation than any of those in U.S states. We also excluded programs without publicly available salary information or cost-of-living data. 616 programs remained, of which 549 were non-unionized, and 67 were unionized. We excluded programs that were not part of a CSA (97 non-unionized programs, 2 unionized programs). For comparisons of vacation time, we excluded programs that did not mention vacation policies on their website or in publicly available sources (22 programs). To assess the impact unionization and contract negotiation had on total compensation, we excluded unionized programs that had not yet negotiated a Union contract (6 programs) or did not publicly disclose union dues. Since unions may be formed as collective bargaining units for multiple programs, each union was treated as the unit of analysis rather than its constitutive programs, yielding a total of 55 bargaining units that were analyzed.

### Compensation definition

For monetary benefits (e.g., Housing stipends, Technology stipends, etc.), we only counted benefits that provided unrestricted funds for the calculation of Total Compensation. For example, a program that provided a cellphone for work use would not be counted for a “Technology Stipend”, while a program that provided $1000 cash for the purchase of technology would. “Other Stipends” were used to classify stipends that are not commonly provided (e.g., Investment stipends, Wellness stipends). We recorded vacation time as either greater than or less than 4 work-weeks of vacation. Stipends for medical boards and certifications were excluded because of high variability in the reporting of these benefits.

### Economic adjustments

Most programs reported salary data for 2023, but some reported 2022 or 2024 salaries. We normalized incomes to 2023 based on a mean annual 2.4% increase in salary [4].

Unionized programs frequently cluster around metropolitan areas with higher costs-of-living [5]. We adjusted incomes for cost-of-living by using the regional price parity index from the Bureau of Economic Analysis, cross matched to program zip code [6]. We summed salaries and monetary stipends, and subtracted Union dues to calculate total compensation.

### Statistical analysis

We compared residency program type (university based, community based, community-based university-affiliated) between non-unionized and unionized programs by Fisher’s exact test.

We employed a multi-level regression model to further control for region-specific factors that influence total compensation. We used presence in a Combined Statistical Area (CSA) as a level for our mixed-effects regression analysis. Residency program type (university based, community based, community-based university-affiliated) affects resident compensation and thus was included as a covariate in the analyses. For continuous variables, we analyzed data using linear mixed-effects regression. For binary variables, we analyzed data using logistic mixed-effects regression. For all statistical procedures, alpha-value was set to 0.05, and p-values were adjusted following controlling for the False Discovery Rate via the Benjamini-Hochberg method (12 tests). All tests were two-sided and conducted in R-4.3.2.

## Results

We analyzed compensation data from 452 non-unionized and 55 unionized internal medicine residency programs. Unionized programs were significantly more likely to be university-based (**Table 1**).

**Table 1:** Geography and affiliations of unionized and non-unionized internal medicine residency programs in the United States.

	Program No./Total No. (%)				
Program Characteristic	Unionized	Non-Unionized	Odds Ratio	95% Confidence Interval	p-value ^a^
**Unionization Status**	55/507 (10.8)	452/507 (89.2)	NA	NA	NA
**Program Affiliation**					
University-based	23/55 (41.8)	98/452 (21.7)	1.93 ^b^	1.08, 3.37	0.04
Community-based University affiliated	28/55 (50.9)	234/452 (51.8)	0.98 ^b^	0.58, 1.63	0.99
Community-based	4/55 (7.3)	120/452 (26.5)	0.27 ^b^	0.07, 0.77	0.02
**Program Region**					
Northeast	29/55 (52.7)	117/452 (25.9)	NA	NA	NA
Midwest	4/55 (7.3)	108/452 (23.9)	NA	NA	NA
South	3/55 (5.5)	170/452 (37.6)	NA	NA	NA
West	19/55 (34.5)	57/452 (12.6)	NA	NA	NA

^a^ Adjusted after controlling for the False Discovery Rate

Compared to non-unionized programs, unionized programs were significantly more likely to provide benefits of at least 4 weeks of vacation time (53 of 55 vs 317 of 430; odds ratio [OR], 11.3 [95% CI 1.97–65.13], p = 0.02), relocation stipends (11 of 55 vs 38 of 452; OR 4.45 [95% CI, 1.03–9.31], p = 0.04), and technology stipends (13 of 55 vs 35 of 452; OR 3.98 [95% CI, 1.66, 9.56], p = 0.008). (**Table 2**).

**Table 2:** Benefits offered by unionized and non-unionized internal medicine residency programs in the United States.

	Program No./Total No. (%)				
Benefit Type	Unionized	Non-Unionized	Odds Ratio	95% Confidence Interval	p-value ^a^
**Vacation Length**					
<4 weeks	2/55 (3.6)	113/430 (26.3)	11.32 ^b^	1.97, 65.13	0.02
> = 4 weeks	53/55 (96.4)	317/430 (73.7)			
**Housing Stipend**	12/55 (21.8)	18/452 (4.0)	1.35 ^b^	0.37, 4.96	0.77
**Relocation Stipend**	11/55 (20.0)	38/452 (8.4)	4.45 ^b^	1.03, 19.21	0.04
**Technology Stipend**	13/55 (23.6)	35/452 (7.7)	3.98 ^b^	1.66, 9.56	0.008
**Sign-on Bonus**	3/55 (5.5)	1/452 (0.2)	NE	NE	NE
**Other Stipend**	8/55 (14.5)	46/452 (10.2)	1.73 ^b^	0.68, 4.41	0.27

Abbreviations: NE: Not estimated

^a^ Adjusted after controlling for the False Discovery Rate

In aggregate, PGY-1 salary was higher for unionized vs. non-unionized programs ($69648 vs. $62214; β = 2108.6, [95% CI 670.7–3563.7], p = 0.002) (**Table 3**). However, after adjusting for cost-of-living, there was not a significant difference in PGY-1 salary between unionized and non-unionized programs ($62515 vs $62475; β = -9.1, [95% CI. -1317.5, 1299.7], p = 0.99) (**Table 3**).

**Table 3:** PGY-1 salary and total compensation offered by unionized and non-unionized internal medicine residency programs in the United States.

	Program Type				
Outcome	Unionized	Non-Unionized	β	95% Confidence Interval	p-value ^a^
**PGY-1 Salary, mean (SD), $**	69648 (6145)	62214 (6069)	2108.6	670.7, 3563.7	0.002
**Total Compensation, mean (SD), $**	73477 (9117)	63251 (6596)	4600.2	3576.16, 5728.2	<0.001
*Following Adjustment for Cost-of-Living*					
**PGY-1 Salary, COL Adjusted, mean (SD), $**	62515 (5669)	62475 (5149)	-9.1	-1317.5, 1299.7	0.99
**Total Compensation, COL Adjusted, mean (SD), $**	65887 (7752)	63515 (5701)	2076.7	607.6, 3551.5	0.03

Abbreviations: PGY: Postgraduate year. SD: Standard Deviation. COL: Cost-of-living

^a^ Adjusted after controlling for the False Discovery Rate

PGY-1 total compensation was higher for unionized vs. non-unionized programs ($73477 vs $63251; β = 4600.2 [95% CI 3576.16, 5728.2], p<0.001). These findings persisted after cost-of-living adjustment ($65887 vs $63515; β = 2076.7 [95% CI 607.6, 3551.5], p = 0.04) (**Table 3**).

## Discussion

Unionized Internal Medicine residency programs are significantly more likely to offer benefits like increased vacation time and stipends compared to their non-unionized counterparts. After adjusting for cost-of-living, unionized programs do not offer higher salaries. However, even after payment of union dues, increased stipend disbursement leads to higher total compensation in unionized residency programs.

This study is the first to investigate the association between internal medicine residency program unionization and compensation. A previous report showed no significant difference in salaries among unionized vs. non-unionized general surgery residency programs [5]. However, internal medicine residency programs are the most common types of training programs, and house staff compensation is often independent of specialty. This analysis captures many of the residency training sites in the United States and presents a broad overview of the impacts of unionization on house staff compensation. Further, our multi-level statistical approach enables the comparison of compensation patterns while controlling for region-specific and affiliation-specific variables that may impact remuneration. This is an especially important point given that the geographical distribution of unionized residency programs is primarily on the coasts (**Table 1**) which are higher cost-of-living regions of the country [6].

Limitations include the cross-sectional rather than longitudinal nature of this study. The number of unionized programs is relatively small, and causal relationships between unionization and the study outcomes cannot be definitively shown. In the future, as more programs unionize, the longitudinal impacts of unionization can be investigated. The eligibility criteria applied in this study enabled a fair comparison of compensation metrics but may have impacts on the generalizability of these findings, particularly in rural programs (not part of a CSA) or those in U.S. territories. One phenomenon that was not accounted for in the analysis was the increase in salaries provided by institutions where residents are threatening to unionize. Additionally, there are several other factors that are often negotiated over in unionization discussions. Other outcomes of unionization, including resident well-being, duty hours, workspace improvement, medical board passing rates, post-residency graduation outcomes, or insurance and retirement benefits, were not investigated.

Unionization efforts amongst residency trainees will undoubtedly continue as economic and lifestyle pressures increase. Reports such as this one which detail characteristics of unionized and non-unionized residency programs can be helpful in guiding decision-making regarding unionization. Unionized residency programs compensate trainees more than non-unionized programs, but non-economic impacts are important consequences of unionization as well. Further research is needed to fully understand the advantages and disadvantages of unionization by physicians-in-training.
